# Ntombi Vimbela! Sexual violence risk reduction intervention: pre and one-year post assessments from a single arm pilot feasibility study among female students in South Africa

**DOI:** 10.1186/s12889-023-16149-x

**Published:** 2023-06-27

**Authors:** Mercilene Tanyaradzwa Machisa, Pinky Mahlangu, Esnat Chirwa, Ncediswa Nunze, Yandisa Sikweyiya, Elizabeth Dartnall, Managa Pillay, Rachel Jewkes

**Affiliations:** 1 South African Medical Research Council Gender and Health Research Unit, Pretoria, South Africa; 2 School of Public Health, Faculty of Health Sciences, University of Witwatersrand, Johannesburg, South Africa; 3 Sexual Violence Research Initiative, Pretoria, South Africa; 4 Department of Basic Education, Pretoria, South Africa

**Keywords:** Sexual violence, Intimate partner violence, Campus intervention, Pilot and feasibility study, South Africa

## Abstract

**Background:**

The extremely high prevalence of sexual violence victimisation reported among female students in South African public higher education demands urgent action to develop, rigorously evaluate and scale effective prevention interventions. This article details findings from a pilot feasibility study of Ntombi Vimbela! a campus sexual violence risk reduction intervention developed to tackle the high burden of sexual violence in higher education institutions in South Africa.

**Methods:**

Ntombi Vimbela! (NV!) is a sexual violence risk reduction intervention that comprises sexuality empowerment, gender and social norm change, early-risk identification, self-defence, resistance and mental wellbeing components. NV! is comprised of ten workshop sessions running for 3.5 h each. Workshops are co-delivered by two trained peer facilitators per group of at most 20 first-year female students. One-year post-intervention quantitative outcome assessments were remotely completed by 98 participants who participated in the NV! pilot workshops. Qualitative assessments were conducted with 35 participants through in-depth telephone interviews (IDTIs).

**Findings:**

One year after attending NV! workshops, most participants reported improved awareness of sexual rights, assertive communication, shifts in gender equitable beliefs, reductions in rape myth acceptance, improved expressed sexual relationship power sexual decision-making, and improved negotiation within their intimate relationships. Participants’ depressive symptoms also significantly decreased. Many participants improved awareness of sexual assault risk and vigilance, including using self-protection strategies such as removing themselves from environments where alcohol intoxication posed sexual assault risks. Some participants used assertive communication to withstand peer pressure to engage in risky sexual behaviours. Most participants scored highly on the self-defence efficacy scale. Some participants were exposed to and successful in using verbal and physical resistance strategies in potential sexual assault risky situations.

**Conclusion:**

These findings indicate the potential beneficial effects of NV! as a campus sexual violence risk reduction intervention at one-year post-intervention, which must be evaluated in a future rigorous randomised control trial.

**Pilot trial registered at:**

ClinicalTrials.gov NCT04607564 on 29/10/2020.

**Supplementary Information:**

The online version contains supplementary material available at 10.1186/s12889-023-16149-x.

## Introduction

Research focused on sexual violence on South African campuses has shown that it is a significant social problem that has negative health and academic outcomes for affected female students. A recent survey showed that about 20% of a sample of female students in selected public university and technical college campuses experienced sexual victimisation in the preceding year [1]. Technical college participants were more vulnerable, reporting a higher prevalence of past-year sexual victimisation (27%) compared to university participants (15%)[1]. Furthermore, the survey identified vulnerability factors for sexual victimisation that have been reported in campus studies elsewhere i.e. being in the first year of enrolment, being younger, having poorer family or community backgrounds, experiencing food insecurity, having prior experience of childhood sexual abuse or intimate partner violence, engaging in risky sexual behaviours, reporting mental ill-health symptoms and harmful alcohol use increased female students’ vulnerability [1–7]. Previous qualitative research conducted on South African campuses also found increased vulnerability to sexual violence among female students who came from low-income households, engaged in sexual risk-taking, including transactional sex or involvement in age-disparate or inequitable intimate relationships [8–10].

However, there is limited evidence on effective sexual violence prevention interventions in South African campus settings which necessitates urgent scholarly work in this area [11, 12]. Strides have however been made to develop and evaluate campus sexual violence interventions in high income country (HIC) university settings [4, 13, 14]. These studies indicate that sexual violence prevention programmes which target female students can increase their knowledge of sexual assault risk, enhance their recognition of personal risk for sexual assault and build their confidence to implement different strategies to minimise, avoid or resist sexual assault [17, 51]. The high rates of sexual assault, our increased understanding of risk factors and the current lack of evidence on effective sexual assault campus based reduction programmes provides a strong rationale and increasing momentum for research that focuses on developing and evaluating interventions that target female students [15–17].

Several sexual violence risk reduction and resistance interventions that target women have been found effective in reducing female students’ experience of sexual violence in high income campus settings [4, 15, 17–20]. The effective sexual violence risk reduction and resistance interventions have been designed to increase women’s recognition of personal risk for sexual assault [17, 21]. They emphasise that early risk detection alleviates sexual victimisation and provide participants with information that helps with early recognition of the risky behaviours of potential perpetrators and contexts in which sexual assault risk is exacerbated [15, 17, 20]. On campus settings, this, for example, includes awareness of alcohol as a common contextual risk factor for sexual assault situations. Participants are provided with knowledge to assist them to develop strategies to reduce risks associated with potential perpetrators’ use of alcohol to incapacitate and take advantage of women [17, 22].

Other aspects of effective sexual violence risk reduction and resistance interventions that assist in reducing women’s risk of sexual victimisation include the empowerment of women by equipping them with skills needed to address emotional and cognitive barriers and building their confidence to use verbal, physiological and physical self-defence or resistance strategies [15, 17, 20]. Several HIC studies have proven that higher self-efficacy and confidence to use physical resistance strategies confers protective effects for sexual assault experience [6].

Sexual intimate partner violence (SIPV) occurs within the context of gender inequitable relationships and women who experience SIPV are at increased risk of experiencing other forms of intimate partner violence [23, 24]. This makes it imperative that interventions aimed at reducing sexual violence must be gender-transformative and be designed to facilitate critical reflection as a means to shift gender beliefs which are a key driver of violence within relationships [16, 17, 23]. Effective campus sexual violence interventions must therefore contain materials that provide sexual empowerment education (i.e., education on sex, sexuality, and sexual rights) to increase participants’ comfort and ability to talk about their sexual rights, sexual preferences, values, sexual practices and communicate assertively about sex and their relationship desires with sexual partners [17, 20]. Through group discussions, participants in programmes providing sexual empowerment discuss and identify: characteristics of healthy sexual relationships, relationship dynamics that infringe on women’s rights and autonomy and critically reflect on sexual violence perpetrated by intimate partners [17]. To be effective, interventions should be gender-transformative, thus including feminist theory, feminist activism and women’s experiences, along with critical engagement on issues to shift inequitable gender beliefs, acceptance of rape myths and victim blaming attitudes [16, 17, 23]. Addressing victim blaming attitudes as part interventions is also a critical ingredient of effective sexual reduction interventions. Victim blaming has been found to lead to self-blame and under-reporting of incidents which in turn fuels the impunity of perpetrators [25, 26].

While campus sexual risk reduction interventions have been evaluated in HIC settings, with fewer interventions proven effective, such intervention work in low and middle income country (LMIC) and Southern African campus settings is emergent [11]. Notwithstanding, scholars have made significant progress in developing and evaluating a repertoire of community level interventions that address violence against women (VAW) in LMICs settings [23, 27, 28]. The lessons learnt and insights from intervention research in LMICs are pivotal in guiding and informing further work to develop, adapt and test campus sexual violence interventions in LMIC settings which are urgently needed to address the high prevalence of sexual violence [1, 12, 29]. This article presents findings from pre and one-year post assessments among first year female students participating in the pilot feasibility study of Ntombi Vimbela! a newly developed sexual violence risk reduction intervention [11, 30].

## Methods

### NV! Development

**Fig. 1:**
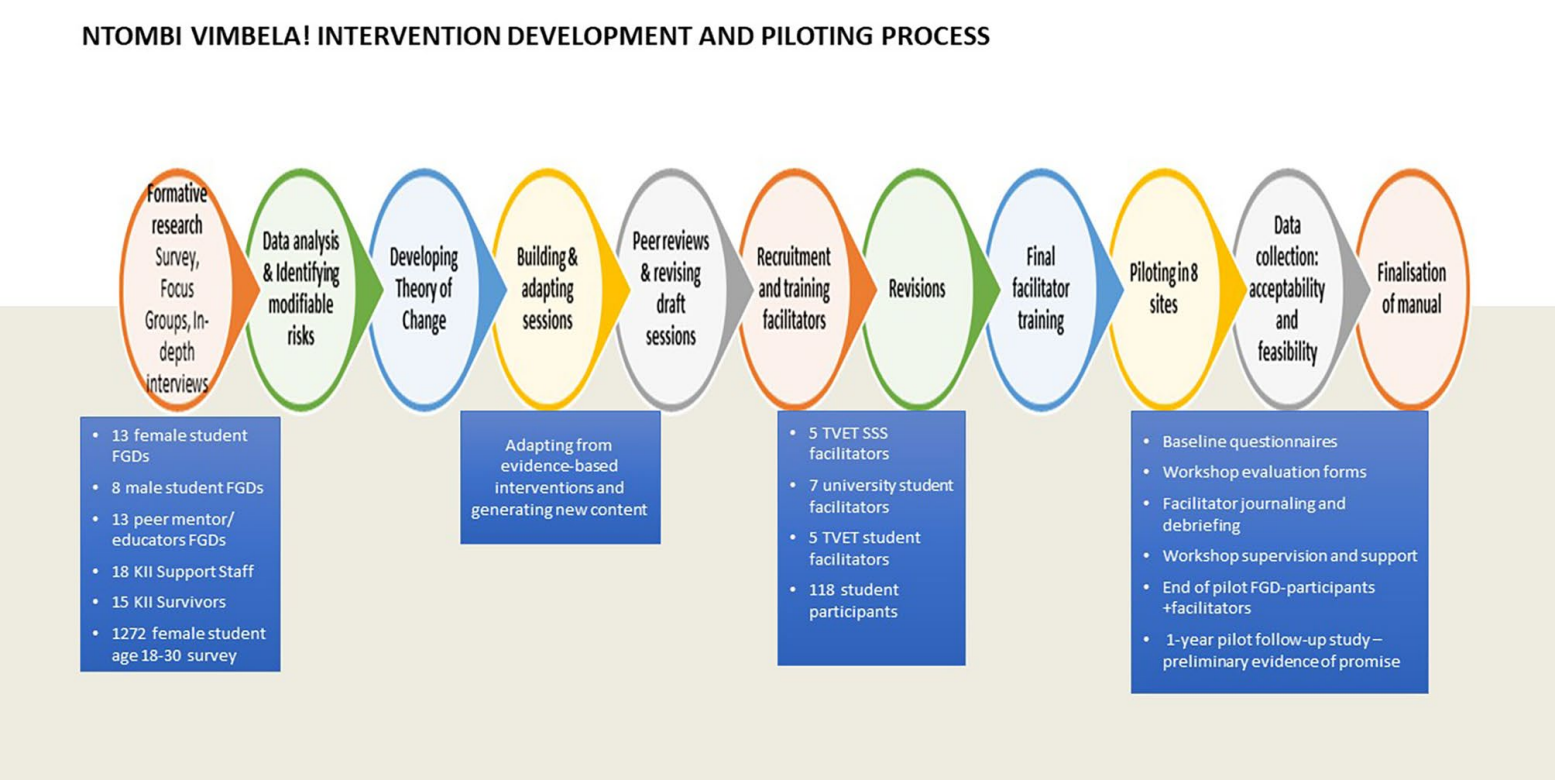
Ntombi Vimbela! Intervention development and piloting process

The steps taken in developing and conducting the Ntombi Vimbela (NV!) single- arm pilot feasibility study were previously published [11, 30]. The intervention development process was guided by the 6SQUID method and also applied aspects from intervention mapping approaches which are frameworks that have been used in the development and planning of health promotion or behavioral change programmes [31, 32]. Figure 1 shows a pictorial flow of steps taken in developing NV! starting with conducting formative research, identifying modifiable risk factors, developing a theory of change, developing the Ntombi Vimbela! intervention manual - which is the change mechanism, testing it first with a group of facilitators and refining before finally conducting a non-randomised pilot feasibility study among groups of volunteering first year female students in eight conveniently selected campuses.

Between 2018 and 2019, we conducted mixed methods formative research aimed to understand sexual violence occurrence and the factors increasing female student’s vulnerability at eight selected Technical and Vocational Education and Training Colleges (TVETs) and historically disadvantaged universities (HDUs) located across five South African provinces namely Eastern Cape, Gauteng, KwaZulu Natal, Mpumalanga and Limpopo [1, 33, 34]. Individual level factors that increased female students’ vulnerability to sexual violence included being first year female, having poor socio-economic family backgrounds, lacking food or other material resources, engaging in risky sexual behaviors including having multiple sexual partners and having transactional sex, reporting mental ill health and harmful alcohol use, limited understanding of sexual assault risky situations including increased vulnerability in social contexts where high levels of alcohol or other substances are present or consumed; acceptance of gender inequitable and victim blaming beliefs ; having less power within inequitable sexual relationships with men; abuse of power and sexual entitlement expressed by male staff and female students’ emotional barriers to practicing assertive communication skills [1, 29, 33, 34]. At the institutional level, poor implementation of sexual violence policy and disciplinary responses fueled students’ lack of trust in available support structures resulting in under-reporting of cases and limited survivor utilisation of services [34].

### NV! Theory of change

**Fig. 2:**
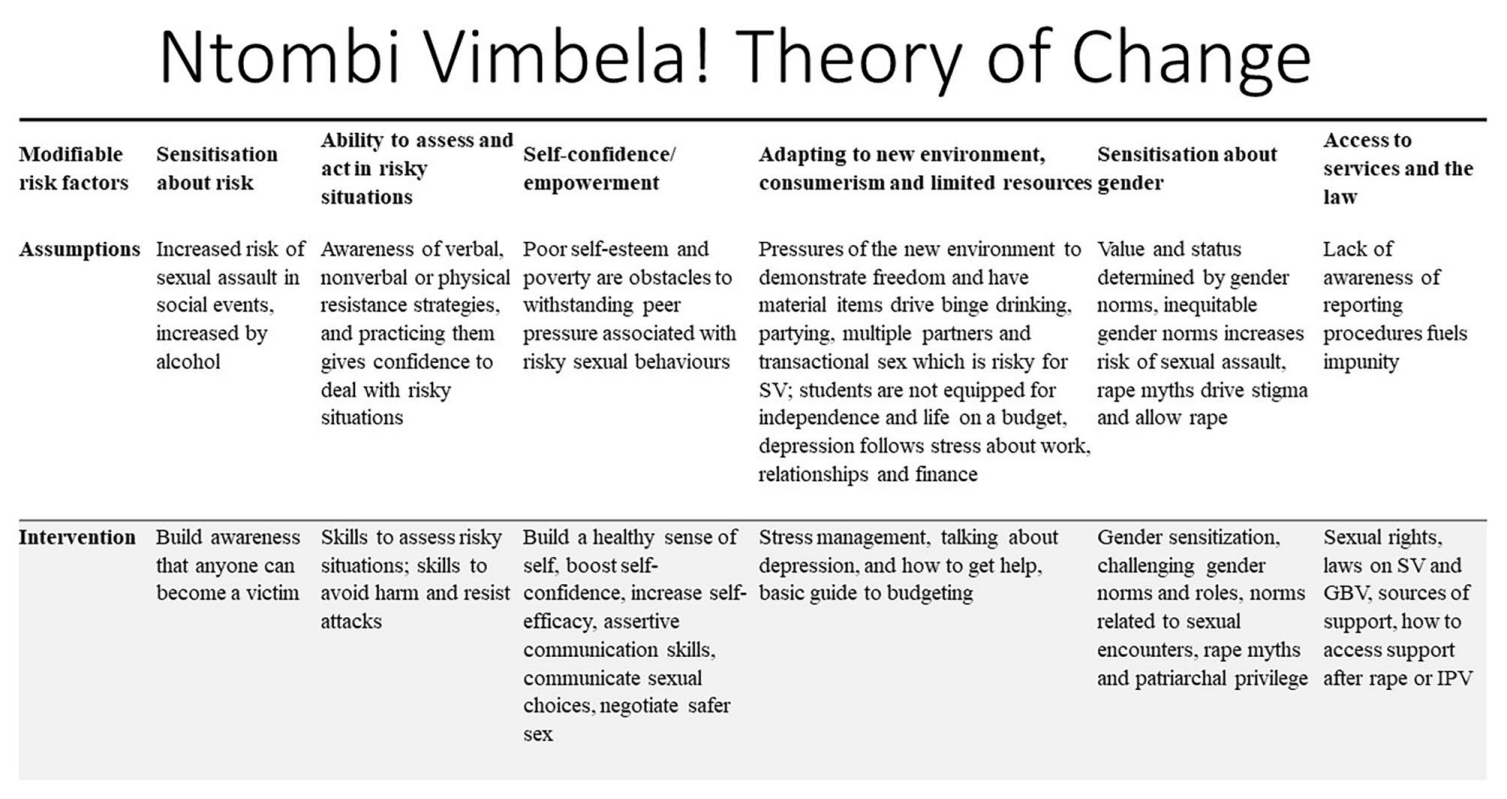
NV! Theory of change

Informed by the formative research findings, we designed and focused NV! on reducing sexual violence vulnerability through addressing the risks encountered by first year students in campuses. The findings about the vulnerability factors also informed NV!’s theory of change, shown in Fig. 2. NV! aims to reduce sexual violence experience by raising awareness about sexual rights, violence against women and girls and its drivers, sensitizing about gender inequality and sexual assault, shifting unequitable gender beliefs, equipping participants with skills to assess and act in situations where there is a high risk of sexual assault, empowerment and enhancing resilience and skills to withstand social and material pressures in college or university, promoting mental health and coping and utilisation of health, psycho-social services and access to justice for survivors, enhancing communication skills and building healthy sexual relationships, and fostering empathy towards survivors [11, 30].

### NV! Co-development and facilitator training

NV! content was drafted and refined through a peer-review process and also incorporating feedback from female students and staff who were recruited to be part of the development phase and became facilitators in piloting the intervention. We tested and refined NV! further through two phases, first, by delivering NV! to a small group of 17 female students and student support staff who were recruited through adverts posted around the campuses to become facilitators and then delivering it to eight groups of female students in different campuses in a pilot. The criteria for recruitment as a facilitator included being a young women ages 18–30; either a student or student support staff member in the selected institution; had passion and interest to learn more about sexual violence and how to prevent it; and who confirmed their availability to facilitate workshops in the pilot phase.

Facilitators were trained on facilitation and interpersonal communication skills, using participatory learning approaches, gender equality and sexuality, managing small youth group dynamics, WenDo self-defense tactics and workshop administrative skills. The first training was held at the SAMRC offices over nine days and aimed to introduce the NV! content to them as participants. After this, they were introduced to and rehearsed the facilitation methods used to deliver NV! content. During this first training, their feedback was collated together with the research team’s observations and used to further revise the NV! manual. The second refresher training was held two months after the first training and over seven days. It was aimed to alert the facilitators to the revisions in the manual, to rehearse delivering NV! and to enhance facilitation skills as well as to prepare them to implement and administer NV! workshops in their campuses.

### NV! Delivery and content

NV! is a sexual violence risk reduction intervention comprised of 10 sessions of 3.5 h each, delivered by two co-facilitators per group, and is described in a standardized manual [30]. It is intended to be facilitated in groups of 15–20 young women by peer women of a similar age (18–30 years), trained and experienced in facilitation. NV! session modalities included participatory group and critical reflection methods to challenge gender inequitable beliefs, enhance personal life skills and stimulate reflection on strategies for sexual violence risk reduction [30]. Table 1 shows a schematic representation of the content and focus of NV! sessions. Session 1 exercises focus on group formation and introducing the intervention to the participants [30]. Sessions 2–5 are designed to promote gender-equitable relationships and to empower participants with skills to improve their relationships through exercises that include reflecting on intimate relationship dynamics, sex and consent, assertive communication [30]. Sessions 4 and 5 exercises challenge inequitable gender norms, rape myths, victim-blaming attitudes and behaviours that contribute to gender inequality, violence and vulnerability for women [30]. Session 6 exercises are designed to promote mental health literacy, enhance participants’ problem-solving and coping skills, provide campus and local services information and promote positive help-seeking which is necessary to manage stress and address mental ill-health associated with adapting to the higher education environment and other life circumstances [30]. Sessions 1, 7, 8 and 9 exercises reflect on personality characteristics and behaviours that are common among potential perpetrators of sexual violence, situations and places in which participants’ risk of sexual victimisation are elevated [30]. For example, isolated places, parties or other social contexts where there is high consumption and intoxication by alcohol or other substances are identified as risky sexual assault situations [30]. As such, the sessions build participant awareness and enhance capability to assess, acknowledge risky situations and react either verbally or physically to mitigate vulnerability or victimisation [30]. Participants are also introduced to, and practice WenDo self defense tactics as suited to various sexual violence risky scenarios [30]. Session 10 exercises identify and reflect on the material, social, and peer pressures that are common among students and enhance life skills including budgeting and managing personal finances [30].

**Table 1:** Schematic presentation of NV! Sessions and content

**1. HERE WE GO! (3.5 h)** Introductions and Group formation. To explain the purpose of Ntombi Vimbela (NV), how it works; what NV involves; To explore participants’ motivations for joining NV and what they hope to achieve with their participation; To introduce explore the concept of safety
**2. LET’S ASSERT OURSELVES (3.5 h)** To enhance communication and practice assertiveness skills. To explore emotional barriers to reacting assertively.
**3. SEX AND CONSENT (3.5 h)** To encourage women to feel comfortable in their bodies, emphasise sexual rights, explore sex and consenting or non-consent, practice assertive sexual communication, overcome emotional barriers to reacting assertively and explore the different experiences of sex. To enhance awareness about contraception, STIs and condoms
**4. WOMEN, MEN AND RELATIONSHIPS (3.5 h)** To enhance understanding of societal expectations for gender (gender norms), explore how participants feel being treated differently from men and encourage challenging norms; To reflect on expectations within sexual relationships and explore some relationship challenges
**5. VIOLENCE AGAINST WOMEN AND SUPPORT (3.5 h)** To sensitize women on the different ways in which women can be badly treated by male intimate partners and non-partners; To explore emotional barriers to reacting assertively; To build awareness on laws, policies and services available for abused women.
**6. MAINTAINING WELLBEING (3.5 h)** To discuss the impact of stress on our health and well-being; To increase awareness of stressors, symptoms of stress and ways of managing stress that are helpful vs. unhelpful; To identify sources of support – both informal and formal within participants’ institution, community and networks.
**7. ASSESSING SEXUAL ASSAULT RISK (3.5 h)** To explore the concept of sexual assault risk, sensitize participants about sexual assault danger cues; To identify men’s attitudes and behaviours that increase the likelihood that they will attempt to sexually coerce or assault women; To share ideas and explore possible strategies to protect/escape from risky sexual assault situations.
**8 & 9 RESISTING SEXUAL ASSAULT (7 h)** To help participants recognise and overcome/reduce emotional barriers to defending oneself physically against sexual assault and ensure safety; To help participants to realise that there are more effective strategies, including forceful verbal and forceful physical resistance/self-defence strategies that can be used to avoid or resist sexual assault; To introduce WenDo self-defence tactics and the principles of “vulnerable points” which can be used to resist sexual assault situations
**10. DEALING WITH MATERIAL PRESSURES (3.5 h)** To build empathy for and self-awareness on social, peer pressures and participants’ need to fit in; To reflect on participant’s spending habits and how these pressures influence them; Practice a budgeting exercise and reflect on necessary vs. unnecessary expense and better manage money.
**CLOSING PROGRAMME**

### Pilot participant recruitment

We conducted the pilot study to assess NV! relevance, acceptability and feasibility of delivery in eight conveniently selected campuses. We used different marketing strategies to publicise the study and recruit participants in the sites - physical posters were put up in key locations on campuses where there was high student traffic, posts were made on social media platforms and groups accessed by students and research assistants, or facilitators visited classrooms and residences inviting potential participants. In each campus, we aimed to recruit 20 first year female students who were ages 18–30, and keen on participating in a sexual violence intervention over 10 weeks. In the sites where more than 20 first year female students expressed interest, participants were randomly selected to participate in the workshops.

### Pilot intervention workshops and facilitator support

Facilitators co-delivered NV! in pairs, in campus venues but outside of the learning programme on days and times agreed with the participants. Only one NV! session was delivered per week and refreshments were served in each workshop. The research team convened weekly debriefing meetings whose purpose was to get facilitator feedback about their experience of delivering NV! content, steering engagements and managing participant group dynamics. During the debriefing meetings, the research team provided support to the facilitators to enhance their understanding of NV! content and facilitation skills.

### Outcomes data collection

The baseline questionnaire was completed by 98 participants in person on electronic tablets in the Research electronic data capture (REDCap) system [35]. Eighty seven participants took part in the remote follow up survey at 1-year post baseline, which coincided with lockdown regulations in response to the COVID-19 pandemic. We resorted to remote data collection because participants were inaccessible and had switched to remote learning as mandated by the national regulations as a measure to contain the COVID-19 pandemic and the disruptions posed by the prolonged enforced lockdowns [36, 37]. Table 2 shows the outcomes measured by the questionnaire which included past year experience of non-partner rape or partner sexual violence assessed using a modified version of the WHO’s Domestic Violence Questionnaire (DVQ) [38]; self-defence self-efficacy assessed using a summative score of eight adapted items of the Self-defence Self-efficacy Scale [39]; personal gender beliefs assessed using the Gender Equitable Men Scale [40]; rape myths acceptance and victim blaming assessed by the Illinois Rape Myths Scale [41]; relationship control and sexual relationship power [42, 43]; sexual communication self-efficacy [44], and self -esteem measured using an adaptation of Rosenberg’s Scale [45].

**Table 2:** Quantitative outcome measures

Anticipated outcomes	Measurement scale	Number and/example of items	Scale reliability
Equitable gender beliefs- Positive change in personal gender beliefs	Gender Equitable Men Scale (41)		Cronbach’s alpha = 0.64 Raykov’s Reliability coefficient = 0.62
Improved sexual relationship power and assertiveness	Relationship control and sexual relationship power (43, 44)	• 11 items • E.g. When my boyfriend/husband and I disagree, he gets his way most of the time. Responses	Cronbach’s = 0.83 AVES = 0.313 Raykov’s Reliability coefficient = 0.83
	Sexual communication self-efficacy (45)	• 12 items • E. g Would you be able to refuse to do something sexual if you didn’t want it? Responses	Cronbach’s = 0.69 Raykov’s Reliability co-efficient = 0.64
Increased awareness and confidence in applying verbal resistance and physical self-defence tactics in risky sexual assault situations, use of verbal resistance and physical self-	Self-defence self-efficacy scale (40)	• 8 items • I know a number of basic hand strike self-defence moves e.g. straight punch, knife hand, hammer fist that I would be able to use if anyone tried to rape me.	Cronbach’s = 0.74 Reliability coefficient= 0.64
Reduction in incidence of sexual violence past year sexual violence experience defence in actual situations	Modified version of the WHO’s Domestic Violence Questionnaire (DVQ) (39)	• Has a current or previous husband or boyfriend ever forced you to do something sexual that you found degrading or humiliating? Did this happen many times, a few times, once or did it not happen? • Have you ever had sex with a boyfriend/husband when you didn’t want to because he physically forced or threatened or pressured you? • Has this happened in the past 12 months?	n/a
Improved empathy towards rape victims, positive change in rape myths acceptance and victim blaming scale scores	Illinois Rape Myths Scale (42)	• 20 items • E.g I think that when a woman is raped, she is usually to blame for putting herself in that situation.	Cronbach’s = 0.85 Raykov’s Reliability co-efficient = 0.84
Improved self esteem	Rosenberg’s scale (46)		Cronbach’s = 0.86 Raykov’s Reliability co-efficient = 0.86

Thirty-five participants who participated in the survey and had attended at least eight of the ten NV! sessions were randomly selected and consented to participate in remote and in-depth-telephonic interviews (IDTIs). On agreed days and times, trained research assistants used a semi-structured interview guide to conduct the telephonic interviews (Supp 1, Additional File 2). Interviews were conducted in English, but participants were allowed to elaborate their responses in their preferred vernacular language. The follow up IDTIs explored pathways for change for the different outcomes including obstacles to change after NV! All IDTIs were audio-recorded and transcribed verbatim and translated into English by the research assistants in preparation for analysis.

### Data analysis

Guided by the evaluation objectives, qualitative data were analysed inductively using thematic analysis [46, 47]. Four researchers read and re-read the transcripts extracting text that was imputed under defined codes developed from the themes explored in the IDTIs. New codes that emerged were also identified from the data. The team jointly discussed and selected quotes that best illustrated the dominant patterns in the data [48].

Quantitative data were analysed in Stata Version 17. We derived additive scores for all scales and binary measures for sexual violence outcomes. Continuous study outcomes (scores) were summarised using mean and standard deviations or median and interquartile range. Binary or categorical variables were summarised using frequencies and percentages. T-tests and their equivalent non-parametric tests were used to compare baseline and follow-up scores. Chi-square tests were used to assess any differences in binary outcomes between baseline and follow up. Cluster-level (campus-level) analysis was done as a sensitivity analysis to assess the robustness of the individual level analysis. All tests were conducted at 5% significance level.

### Ethics

#### Ethical approval

was received from both the South African Medical Research Council, Research Ethics Committee (EC002/2/2018) and the Department of Higher Education and Training. The pilot trial was registered at ClinicalTrials.gov (NCT04607564) on 29/10/2020. Research assistants sent short message service (SMS) texts and followed up with telephone calls to invite participants to be part of the follow up study via. They asked the participant’s preference for remittance of the full study information sheet between WhatsApp, SMS or email. Participants returned a signed copy of the consent form before participating. All participants were assured that the information they provided in the study would be handled confidentially and that the findings of the study will be reported with complete anonymity. All data were uploaded and stored on the secure server where data access is restricted to only the researchers. The research assistants gave participants lists of GBV support service providers on and close to their location at the time of the study. Participants were reimbursed with (South African Rand (ZAR) 50) cash vouchers and received data (ZAR 15) to remotely access and complete the follow-up survey questionnaire.

## Results

Table 3 shows the participant characteristics. More of the students were enrolled in Technical Vocational Education and Training Colleges (TVETs) (74%), 49% were living in campus residences, 97% were recipients of the government student grant (i.e. National Student Financial Aid Scheme (NSFAS) or other bursary sponsorship, and 81% reported that they would find it difficult to find money in case of an emergency. Less than a fifth of them perceived that they were better off financially compared to their peers.

**Table 3:** Baseline sample description

	All (N = 98)	University(n = 25)	TVET(n = 73)
	N (%)	N (%)	N (%)
**Residence**			
Family Home	20 (20.4)	6 (24.0)	14 (19.2)
Campus	48 (49.0)	13 (52.0)	35 (48.0)
Rented accommodation	18 (18.4)	4 (16.0)	14 (19.2)
Other	12 (12.2)	2 (8.0)	10 (13.7)
**Earned an income in past year**	21 (21.4)	4 (16.0)	17 (23.3)
**Difficult to find money**	79 (80.6)	19 (76.0)	60 (82.2)
**Financial status**			
Better off than peers	16 (16.3)	6 (24.0)	10 (13.7)
Same as peers	52 (53.1)	10 (40.0)	42 (57.3)
Less well off than peers	30 (30.6)	9 (36.0)	21 (28.8)
**Receiving NSFAS/other scholarships**	95 (96.9)	25 (100)	70 (95.9)
**Currently in a relationship**	76 (77.6)	19 (76.0)	57 (78.1)

Table 4 shows statistically significant positive changes in rape myths acceptance, gender equitable beliefs, sexual self-efficacy, and depressive symptoms (p < 0.05). While the changes in sexual relationship power and self-esteem were non-significant – which are in the anticipated direction of positive change. The reduction in sexual violence experience was not statistically significant.

**Table 4:** NV! pilot quantitative outcomes

	Baseline (N = 98)			12 m (N = 87)		
	mean/n	sd/%		mean/n	sd/%	p-value
Rape myths score (low = less victim-blaming)	35.5	8.2		32.6	7.7	0.046
Gender attitudes score (high = more equitable)	25.6	3.5		26.7	2.8	0.026
Sexual self-efficacy (high = good)	40.4	5.2		41.9	4.1	0.028
Depressive symptom score (high = more depressed)	21.3	12.3		16.9	10.4	0.010
Self-esteem score (high-high self-esteem)	32.2	5.9		33.5	4.6	0.095
Relationship control score (high = partner less controlling)	32.1	5.4		33.4	4.1	0.112
Experienced Sexual IPV in past 12 m (%)	13	13.3		7	8.1	0.254
Experienced any sexual violence past 12 m (%)	25	25.5		19	21.8	0.558

### Qualitative findings

#### Confidence to verbally and physical resist against non-partner harassment

NV! sessions focused on sexual assault risk reduction and provided information that participants can use to identify risky sexual assault situations, skills to resist unwanted sexual advances, using assertive language, as well as physical self-defence training. Some participants in the qualitative interviews at one year follow-up reported that they had found themselves in situations where they had applied the skills they acquired from NV! to diffuse situations in which they were at risk of sexual violence. Their narratives showed that participants were able to identify the behaviours of potential perpetrators from their communities and who they were not intimately involved with. They demonstrated confidence to respond verbally and to assertively communicate their disinterest to perpetrators; and when the men ignored them, they screamed to get the attention of other people or bystanders. For example, one participant shared, *“So, my friend and I normally go to this other internet café and the guy there was doing some nasty things to me when I ask for his assistance, but I was able to defend myself. He would touch me and make comments about my beauty, so I told him to stop. Firstly, I told him to stop as I don’t like what he is doing. He did not stop so I screamed and other people looked at him and saw what he was doing, so he stopped. Yes, because he is much older than me. Yes, he stopped!” (TVET participant)*.

Some of the participants reported that they used physical self-defence tactics to push back against unwanted sexual advances and physical assaults by men in public spaces. For example, one participant shared that she had pushed away an older man while taking a taxi ride, “*Like I was telling you about that old man in the taxi, so I pushed him away and it was clear to everyone that I am angry. It was easy (Laughing). You just use your elbow, and you just break the silence”,(TVET participant).* Another participant shared using the physical self-defence tactics to defend herself from a man who was grabbing her “*I was walking on the streets and then came this guy who propose me a lot from home and then he just grabbed my hand and was asking why I don’t talk to him. I told him that you know what I already told you that I don’t like you and I do not want to talk to you. I was trying to walk away so he grabbed my hand and that`s where I applied that hammer fist technique to say get off me.” (University participant).* The quote above reflects that in the described scenario, when the participant recognised threat in the behaviour of an acquaintance who made frequent sexual advances towards her, she used assertive verbal communication to convey her disinterest, and used physical self defence skills to release herself from his hold.

### Confidence to use physical self-defence tactics with intimate partners

Some participants also shared that they calculatedly used physical self defence tactics when they had altercations with their boyfriends. For example, one participant reported an incident where she assertively communicated her displeasure and used physical tactics when he forcefully grabbed her. This was an unexpected reaction to her boyfriend. She shared:

*“P: There’s this time my boyfriend grabbed me forcefully, I made the fist and managed to release the hand he was grabbing, and I stepped away from him, I also told him that I did not like what he was doing. He asked if I am now fighting back at him, and I told him that no I was just releasing my hand from him as I did not like the way he was grabbing me.*

F: Alright. Was it challenging to do that (the fist)?

*P: No, I think I was in a good space, and I was well balanced, and it was the first time to do that except for the time I was learning about it during NV!. (TVET participant)*

### Improved assertive communication, negotiation skills and shifts in intimate relationship dynamics

Some participants applied their newly acquired skills learned from NV! to negotiate power in their relationships through asserting their autonomy and confronting relationship problems. They shared that their relationships changed because the information that they received from NV! sessions enabled them to take back their power and gave them a voice to assertively communicate their sexual rights including becoming more comfortable to talk about sex with their partners:

*“P: Sometimes, he would do sexual things I didn’t like. I would not know what to say and how to respond but I would just keep quiet. But after I attended NV! workshop, I decided that I will have to stand up for myself and how I feel, so I told him that I don’t like the things you do and always say to me. You must stop it.*

F: How did he take it?

*P: He apologised, and we resolved the issue and it never happened. Now, when one of us wants something, we just talk as adults and we agree, we don’t force each other. We talk about how things should be when we are having sex. So, I was telling him what I like, and he told me how he likes things to be done and we are both happy now.” (TVET participant)*

In many instances, participants reflected that broader relationship changes had occurred due to enhanced assertive communication and negotiation skills gained after attending NV! which extended beyond sexual communication to decision-making within intimate relationships. One participant reported, “*Now, I want to be involved in whatever he wants to do in our relationship, he must not decide alone about how things should be done, he must first ask me, and I must agree if I want to.” (University participant)*.

Whilst reflecting on the changes that occurred in their intimate relationships after attending NV!, some participants delved into relationship factors that previously made it difficult for them to assert themselves with their partners. Some of the participants spoke about how they were involved in sexual relationships with older men who materially provided for them. Others previously embraced traditional gender norms that prescribed inequitable dynamics through which they felt they needed to be submissive and such beliefs disempowered them. One participant who overcame these previous barriers reported:

*“I used to find it difficult to stand my ground and argue with my partner, when he says I won`t do a certain thing then I don’t do it because he is a man and he takes care of me financially so why should I say no including sexual things, I thought I should always agree with him. But the NV! workshops taught me that it doesn`t matter if he is taking care of me or what, it`s about me and we need to understand each other, love each other and respect each other and also respect each other`s decisions. Now, we both decide on what to do and we communicate very well like he let me say what I want, and I let him say what he wants and then we decide on what to do. When we are together, I am able to tell him when I do not like a certain thing and if he does not listen, I am able to be bold so that he can see that I am serious about that. (TVET participant).* This quote is evidence of someone who demonstrates having power and voice in their relationship, improved communication skills and shift in gender beliefs.

Some participants shared that they were dissatisfied in their relationships at the time they participated in workshops, but NV! increased their awareness of their rights, helped them to re-evaluate their relationship goals and empowered them to conjure confidence to confront their partners about their relationship challenges. One participant shared,

*“NV! helped me on that side because I used to keep quiet whenever there is something that I don’t like, it would hurt me inside but say nothing and I would tell myself that I am not going to say anything I am just going to let it be but ever since I attended the NV! workshop, at least now I have ways of approaching him, and I now know how to respond to his acts. He used to not respect me, so this other time I decided to tell him that I don’t like what you are doing to me, and he asked what it is it? I told him about the disrespect he has towards me, and I don’t know if it’s the matter of age or what but I don’t like the disrespect. He was shocked because he knew that wasn’t me. So, he sat down looked at me and he asked how long I have kept that. I just said I don’t like it he should stop, then he apologised, and he promised that he will never do it again.” (University participant).* The quotes presented in this sub-theme show how NV! workshops empowered female students with skills they used to improve their intimate relationships by assertively communicating to their partners about the behaviours they did not like and negotiating for their needs to be met.

### Shifts in gender beliefs and exiting abusive relationships

Some participants reported that they felt that NV! empowered them to consider ending the abusive relationships. NV! created a platform in which participants conversed about the negative impacts of intimate partner violence and the protection available for survivors through accessing the justice system and reporting perpetrators to police. Notably, the period of NV!’s pilot coincided with media reports on femicides of young women and students which became the subject of discussion in workshops and WhatsApp groups created to assist planning for workshops [49, 50]. One participant exited a relationship she had with a physically abusive boyfriend. She reflected and reasoned that exiting the relationship possibly stopped her from becoming a femicide victim:

*“P: The last time I saw my boyfriend, he was drunk, and he was carrying a knife, he came to my place at night, and I thought to myself that he will never stop this, it’s better if I end the relationship with him before I am the next one on the statistics of girls killed by their boyfriends. I did not like it so I decided to leave him alone so that I can live my life and focus on my studies. I told him that he did this for the first time, for the second time, and now it was his third time, so because he has done this for too long, I told him it is better if we no longer see each other and break up for the sake of my life and my sister`s safety because he was also aggressive and he once showed me another side of him that I did not know.*

*F: Mm.*

*P: He did not want to accept that but I couldn`t change my decision…He did not like my decision because he was always following me, but I had already decided to leave him because I was also looking at what is happening in our country about girls being killed by people, they are in a relationship with, but I managed to leave him. I was not shy or scared to do that even though he was following me, I told him that if he doesn’t back-off I will report him to the police. He has been quiet now even though I wouldn`t be sure of what he is thinking right now.” (TVET participant)*

Whilst the participant’s account reflects potential risks of backlash from the estranged boyfriend, she demonstrated improved knowledge about the protection she could obtain by reporting to the police.

### Improved vigilance and strategies to minimise sexual assault risks

NV! provides information that helps women to recognise the risky behaviours of perpetrators and contexts in which sexual assault risk is exacerbated. Most participants reported that their awareness of sexual assault risk improved, they became more vigilant and used self-protection strategies for example avoiding isolated places at night or getting lifts from strangers. Some quotes we heard from participants that reflected increased vigilance include:

“… *before the NV! I was not that cautious about things like to always check if there is anyone following me, I was not paying attention to other things but now I am aware and I am very cautious, …. I am more aware than before” (University participant).*

“*I don’t even accept a lift. When a man I don’t know offers me a lift, I just say no thank you. Just a straight no that I don’t want the lift.” (TVET participant)*

*“NV! was very helpful. Especially like if I have to take a taxi at night, I do check if it is mostly males inside because I don’t feel comfortable inside a taxi with most males because they might pretend to not know each other yet they are up to something” (TVET participant)*

*“NV! helped me because I am now very cautious and I don’t go out at night and I avoid dark spaces, but when I do, I always carry pepper spray.” (University participant)*

*“NV! has been helpful because I have learned that as a person you must always be cautious especially when you are on the road at night, you must always look around and check if there is anyone following you and I have learned that walking alone in a dark place is dangerous and that when you go out with someone you must alert the people you know and give them address. (TVET participant)*

NV! content also raised participants’ awareness about alcohol intoxication as a risk factor for victimisation. During workshops, participants critically reflect and discuss strategies for minimizing alcohol-related risks. Participant feedback in the follow-up interviews shows that many of them confidently resisted pressures they got from their peers to engage in risky behaviours. They also avoided or removed themselves from environments where alcohol intoxication posed sexual assault-related risks. One participant shared that because of the lessons she got from NV! she refused to go to an unknown place with a group of men she and her friends just got acquainted with when they went out drinking:

“*So there was this other time where we were out with my friends, and we had fun dancing to the music. There were like 4 to 5 guys who came and asked to join us and we were ok with that. They bought more drinks not only for them but for us as well, we were surprised because we bought our own drinks. Even the conversation now started changing, they were like yah guys there`s this new place over there we should go and check it out. We said no we are not going anywhere with them. And we did not go because now we didn’t know them and didn’t know what they will do when we got there and why they suggest that we change the spot all sudden*.” (*TVET participant).* This quote also reflects that participants did not experience NV! as deterring them from their social lives. Rather, they shared learning from NV! workshops among their peer groups around the behaviours of potential perpetrators, sexual assault risk and practiced vigilance when they detected risk.

## Discussion

This paper presents findings implicating the potential benefits of the Ntombi Vimbela! intervention at one-year post the implementation of a single arm pilot feasibility study conducted among first year female students on South African campuses. The study findings indicate promising evidence of NV!’s benefits that adds to previous preliminary post-intervention qualitative data that demonstrated that NV!’s content was relevant, acceptable and the delivery methods were feasible among first year female students who have sex with men studying on South African campuses [11]. Positive outcomes deduced from the follow-up study’s quantitative assessments include reduction in depressive symptoms, improvements in sexual self-efficacy, positive shifts in victim blaming and gender equitable attitudes and reduction of sexual violence experience. The qualitative assessments indicate that some female first year students who attended NV! workshops benefited from their improved awareness of personal risk for sexual assault, were able to recognise sexual assault risk situations or perpetrator behaviours and had the confidence to appropriately apply self-defense tactics. Participants explained these as pathways that contributed to their perceived success at removing themselves from situations involving male partners, acquaintances, and strangers they judged as risky and their reasoning that this decreased their risk for victimisation. The results emanating from the assessments are consistent and in the anticipated direction that has been reported from the evaluations of effective evidence-based campus and sexual violence risk reduction interventions in other, mostly HIC settings [6].

The findings suggest that after attending NV! workshops, participants improved their knowledge and capability to assess situations that could increase vulnerability for sexual victimisation, and these were often described as social situations where alcohol was present or consumed in high quantities. They explained that practicing self-protective behaviours helped them navigate situations where they perceived themselves vulnerable to intoxication and sexual victimisation. Evaluations of other sexual violence risk reduction interventions have found that when women can acknowledge that situations where alcohol is present or consumed exacerbate sexual assault risks, they are more likely to avoid, remove themselves or devise protective strategies such as limiting their consumption to avoid intoxication and minimise vulnerability to sexual assault [4, 17, 22]. Furthermore, it has been shown that women’s vigilance and increased ability to identify behaviours in potential perpetrators, who are either acquintances or strangers, as “risk cues” can significantly reduce their vulnerability as “easy victim targets” [51].

There has been much scholarly debate around the harms and benefits of implementing interventions that train women on self-defense skills [15, 51]. Antagonists have argued that training women on self-defense places responsibility on them to prevent their victimisation, can insinuate self-blame among sexual violence survivors and that resisting perpetrators may further endanger women [4, 15, 17, 51]. However, the findings presented here align to the proponents in the debate by reflecting some women’s confidence and attempts to verbally resist potential perpetrators and appropriately apply self-defense tactics that they perceived as beneficial to reducing their victimisation by male intimate and non-partners. These positive findings could be attributed to the design of NV! that integrates content aimed to empower women, shift victim-blaming attitudes by emphasising that blame should be apportioned to the perpetrators of violence with resistance skills. Such intervention design has yielded positive outcomes with several interventions for example the EAAA program implemented on Canadian campuses [16–18].

Findings from this pilot-follow up study also show NV!’s promise as a sexual empowerment education and gender transformative intervention. The study findings indicate potential enduring attitudinal and behavioural benefits of NV! that impacted on intimate relationship dynamics and intimate partner violence. Participants explained pathways from positive shifts in gender equitable attitudes, improved assertive communication skills to increased sexual relationship power and voice in intimate relationships as well as challenging less desirable sexual and other relationship dynamics after going through NV! workshops. Shifts in gender equitable beliefs and ted reduction in rape victim blaming and myth acceptance have been identified as primary outcomes in other campus sexual empowerment interventions [6, 16, 17]. However, other scholars have critiqued campus interventions that show success in shifting women’s gender attitudes, reducing rape-myth acceptance and increased knowledge about sexual assault but have not established the durability of attitudinal changes nor established whether the shifts reported are associated with reduced rates of victimization [52]. Notwithstanding, the findings reported from NV’s pilot follow up are unique in that they demonstrate qualitatively the pathways from shifts in gender beliefs to more equitable relationships and reduction in intimate partner violence.

As much as NV! and other women focused sexual violence risk reduction interventions implemented to small groups have shown benefits, it is pertinent to acknowledge that they do not suffice in of themselves to reducing the prevalence of sexual violence on campuses. It is important to acknowledge that the problem of sexual violence on campuses is perpetuated through campus climate and culture that tolerates its occurrence through the lack of or poor implementation of anti-sexual violence policies, protocols, and practices [4]. Moreover, the effective implementation of other institutional policies such as security controls and alcohol policies is pertinent for addressing the problem of sexual violence among students [4].

Less traction will be made by sexual violence prevention interventions on South African campuses without the direct involvement of male students in other individual or relational level violence perpetration prevention programs applying strategies such as by-standerism, alcohol or other substances harm reduction or social norm change, gender transformative and behavioural interventions [4, 13, 16, 17]. While this may be the case, it is noteworthy that globally, interventions to reduce men’s perpetration of campus sexual assaults have lagged and few have been proven effective [4, 13, 17]. Work on men’s gender transformative and violence prevention interventions for South African campuses is emergent but must be expedited to complement the benefits of women’s focused interventions such as NV! in turning the tide of the high prevalence of sexual violence and associated risk factors [33].

Our pilot study was limited by a small sample size, a non-randomised design and there could have been bias among female students that self-selected to participate in the NV! pilot. While we have reported enduring outcomes of change one year post intervention among a small sample of pilot participants without a control group, NV! effectiveness and pathways to change can only be confirmed through evaluating it in a randomised control study that recruits a larger, fully powered sample and measure outcomes quantitatively and qualitatively. This will be the focus of the research team’s future research agenda.

Our work also reflects on the feasibility of collecting violence related data among cohorts of tech-savvy young people in African campus settings by employing remote and digital methods. Scholars in the field have been skeptical about the safety risks that are elevated when participants living with abusive partners are exposed to remote data collection methods such as surveys and IDTIs [53, 54]. However, in our study these risks may have been minimal because the intimate relationships of participants were commonly with male students and other men living in the vicinity of their campuses [11, 33]. Participants were more likely to be living with their families than with their partners during the COVID 19 lockdowns. Notwithstanding, participant privacy was ensured by having the IDTIs conducted at times agreed by the participants where they anticipated minimal distractions from others. The feasibility of participants navigating a structured questionnaire and providing good quality data remotely in some respects was uniquely possible against their academic background of digital remote learning and ownership of devices necessitated by the Covid-19 pandemic [36, 37]. However, such methods may as well apply to the collection of data in large cohort studies where participants may have digital literacy but may no longer be living near the research sites. Even so researchers conducting remote data collection must prioritise participant safeguarding [53, 54].

## Conclusion

The findings presented in this paper add to the preliminary evidence of the enduring benefits of NV! one year post intervention pilot based on individual participant reports gathered through remote data collection methods. Previously, participant groups reported that NV! empowered them with skills to assess and deal with sexual assault risky situations, changed their gender beliefs, shifted their acceptance of rape myths and beliefs, improved communication skills and enhanced self-esteem [11]. The present findings show individual benefits and the application of skills enhanced through participation in NV! workshops. Participants reported reductions in rape myth acceptance, depressive symptoms, engaging in risky sexual behaviours and non-significant reduction in past year sexual victimisation. Other benefits included improved awareness of sexual rights, assertive communication, shifts in gender equitable beliefs, sexual relationship power, sexual decision-making, negotiation within their intimate relationships. Sexual assault risk was reduced through participants’ improved self-defence efficacy, awareness of sexual assault risk, vigilance, avoiding alcohol intoxication and applying verbal and physical resistance strategies. Altogether, the findings show that NV! holds promise as a campus-based intervention for sexual assault risk reduction among female students in South Africa and as such, further rigorous testing in a future adequately powered randomised control trial of NV! is warranted.

## Electronic supplementary material

Below is the link to the electronic supplementary material.

Supplementary Material 1

Supplementary Material 2

## Data Availability

The authors have made the minimal quantitative dataset available as supplementary material to this submission: Additional File 1: NV! Pilot Minimal Data Set.

## List of abbreviations

IDTI: In-depth telephone interviews
HIC: High Income Country
LMIC: Low- and Middle-Income Country
NSFAS: National Student Financial Aid Scheme
NV!: Ntombi Vimbela!
SMS: Short Message Service
SIPV: Sexual Intimate Partner Violence
TVET: Technical Vocational Education and Training Colleges
VAW: Violence Against Women
ZAR: South African Rand
