# Supplementary material for: Ntombi Vimbela! Sexual violence risk reduction intervention: pre and one-year post assessments from a single arm pilot feasibility study among female students in South Africa
Source: BMC Public Health. 2023 Jun 27;23:1242. doi: 10.1186/s12889-023-16149-x (PMC10294310; doi:10.1186/s12889-023-16149-x)
Supplement: Supplementary file 2 — Supplementary Material 2 [file 12889_2023_16149_MOESM2_ESM.docx]

***Supp 1 - Excerpts of follow-up in-depth telephone interview guide***

1. **Communication- parents, partners & peers**
2. Can you tell us how your communication is with your parents/guardians since you attended NV workshops? Can you share with us a story or examples of instances where you were communicating with your parents/guardians and used what you learned from NV workshops, if any? How have they received it?
3. Can you tell us about your communication with your intimate partners since you attended NV workshops? How have you been you able to ensure that you communicate your interests or preferences (what you want or not want including sexual things) and how did that go?

**Probe**: Please share with us examples of how you succeeded or had challenges when you were trying to communicate your interests or preferences to your partner.

1. Can you tell us about your communication with your peers since you attended NV workshops? How easy or challenging has it been for you to communicate your interests or assert yourself when you are pressured to do something you don’t want? How did that go?
2. How has the information and skills about communication from NV helped you to communicate with parents, intimate partners and peers in the context of Covid-19? Can you share stories.
3. **Risk and sexual violence**

How has gaining information about risk cues through NV been helpful or not in your life?

1. Since we learned about risk cues in the NV workshops, could you tell us about a time where you were able to use the information or skills you got. Can you give us specific examples?

**Probe:** What did you do?

1. **Gender attitudes**
2. How has gaining information about gender through NV been helpful or not in your life?

Probes

How has it changed how you view relations between men and women? How has it changed how you view power between men and women?

1. How has been the reaction of your parents, intimate partner(s) and peers when you shared or practised the learning about gender and power?
2. How has learning about gender and power influenced how you now relate with your intimate partner(s).

**Probe** How has the learning about gender and power shaped your practices in their intimate relationship(s). Please share about this in terms of making decisions, how and when sex is done, contraceptives, sexual and reproductive health, bodily autonomy.

1. How has learning about gender and power influenced how you relate to men in general?
2. **Resistance- verbal, nonverbal, physical**
   1. How has gaining information and skills about sexual assault resistance (verbal and non-verbal/self-defence) through NV been helpful or not in your life?

**Probe:** Can you share with us stories where you were able to use any of the sexual assault resistance strategies. How did that go?

**Probe:** Can you share with us instances where you experienced challenges in using the sexual assault resistances strategies? Why do you think you experiences those challenges?

1. **Barriers to implementation overall**
2. What are the things that you have learned in NV that you are finding hard to implement in your life and relationships and why?
3. What do you think were the reasons you found certain things (e.g. skills and information) you learned in NV difficult to implement in your life?
